# A hypoplastic patella fracture in nail patella syndrome: a case report

**DOI:** 10.1186/1752-1947-6-196

**Published:** 2012-07-16

**Authors:** Shane C O Neill, Colin G Murphy, John P McElwain

**Affiliations:** 1 Department of Orthopaedics, The Adelaide and Meath Hospital Incorporating the National Children’s Hospital, Tallaght, Dublin, Ireland

**Keywords:** Nail patella syndrome, Fracture, Patella, Osteo-onychodysplasia, Aplasia

## Abstract

**Introduction:**

Nail patella syndrome is a rare autosomal dominant hereditary condition, with an incidence of 22 per million in the United Kingdom. The syndrome’s most common features include iliac horns, hypoplastic patella and nail dysplasia.

**Case presentation:**

We report the case of a 26-year-old Caucasian man with nail patella syndrome who sustained a fracture of his right hypoplastic patella after a fall. His right knee became swollen and he was unable to extend against gravity immediately post fall. Radiographs revealed a fracture of the lower pole of his right patella with associated complete disruption of the extensor mechanism of the knee. He underwent operative fixation and his post operative course was uneventful. He was further treated post operatively with a full knee cast and graded immobilization. At six months he had regained the full range of motion at the knee joint.

**Conclusions:**

To the best of our knowledge, this is the only case report in the literature describing a patella fracture in an individual with nail patella syndrome. We hypothesize that given the extent of pre-existing knee joint impairment in these individuals, functional outcome may be inferior, suggesting the need for more frequent follow-up.

## Introduction

Nail patella syndrome (NPS) itself a rare condition, has been relatively well described in the literature. However, a traumatic patella fracture in this patient cohort has not yet been reported. We present the case of 26-year-old man with this condition who sustained a patella fracture.

## Case presentation

A 26-year-old Caucasian man with nail patella syndrome presented to the accident and emergency department, after slipping on a step while running and sustaining a twisting injury to his right knee. The right knee became swollen and was painful on attempted weight bearing directly after the fall. He had no other associated injuries.

His past medical history was remarkable for a diagnosis of osteo-onychodysplasia or NPS, which was discovered at the age of 11 after noting bilateral thumb nail changes. Anterioposterior pelvis radiography performed at initial diagnosis also revealed bilateral iliac horns, a finding pathognomonic of NPS. He had normal elbow function and yearly renal and ophthalmological investigations since his diagnosis have been normal. He reported no previous patella dislocation, but had complained of mild knee pain after extensive exercise in the years prior to the injury.

Initial laboratory investigations on this admission were all within normal limits.

**Figure 1:**
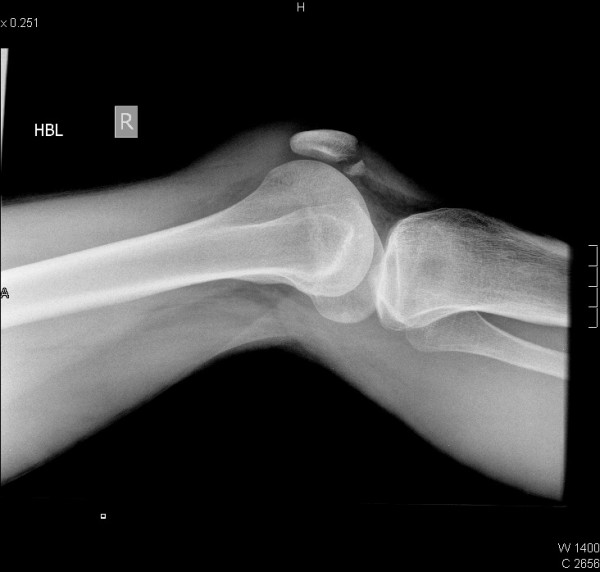
Lateral view of the right knee showing an avulsion fracture of the lower pole of the patella.

**Figure 2:**
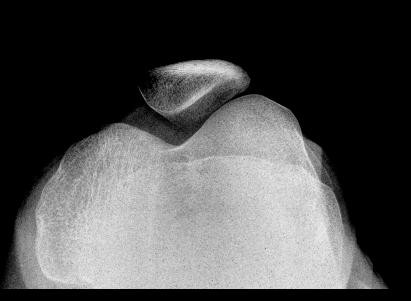
Skyline view of the knee showing a hypoplastic patella.

On examination his right knee was swollen, clinically consistent with hemarthrosis and was tender over the lower pole of his right patella. He was unable to extend his right knee against gravity. Bilateral dysplastic thumb nails were noted. Other fingernails and toenails were normal. Radiographs revealed a fracture of the lower pole of the right patella (Figure 1). Bilateral hypoplastic patella and a hypoplastic left medial femoral condyle were also incidentally noted, consistent with his existing diagnosis of NPS (Figure 2).

As the extensor mechanism was disrupted, surgical management was indicated. A lower midline incision was made, and the hemarthrosis evacuated. A full thickness tear of the lower patellar retinaculum was noted, and on closer inspection of the fracture site, the distal fractured fragment was seen to be small and almost completely extra-articular. His bone quality was good. The distal fragment of his patella was excised, and a retinacular reconstruction undertaken with a non-absorbable, braided, polyethylene suture (2 Ethibond), followed by a layered closure.

His post operative course was uneventful and he was discharged with an above knee full cast and analgesia. The cast was removed after two weeks and a hinged knee brace applied, allowing 0° to 30° range of movement for a further two weeks. Flexion was increased to 90° four weeks post operatively and at six weeks all immobilization aids were discontinued. A Dual Energy X-ray Absorptiometry (DEXA) scan was performed two months post fracture, which revealed mild osteopenia at the right femoral neck; Z Score −1.7. No previous bone mineral density (BMD) measurement had been taken prior to this.

**Figure 3:**
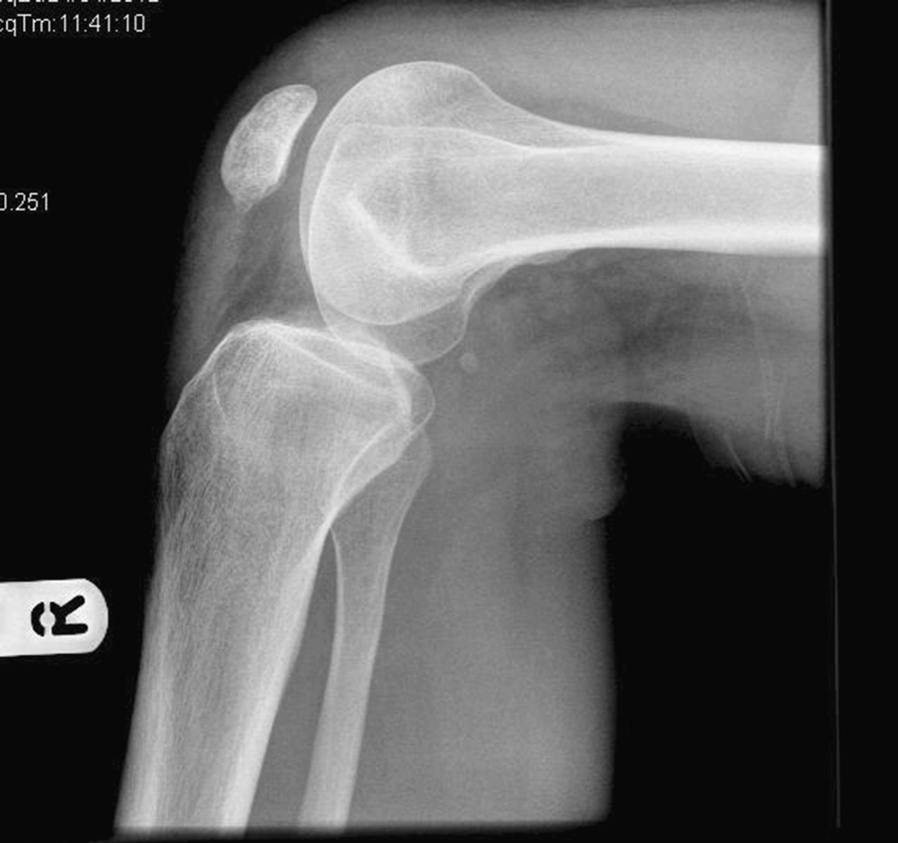
Lateral view of the right knee six months post injury.

At six months follow up, his full knee range of motion had been restored and he had returned to work successfully. On functional assessment he scored 100 on the WOMAC score [1]. A lateral X-ray of his right knee revealed no residual evidence of fracture (Figure 3).

## Discussion

NPS is a rare autosomal dominant hereditary condition, also known as Hereditary Osteo-Onycho Dysplasia (HOOD) or Fong’s syndrome [2]. The condition exhibits variable expression, attributed to mutations of the LMX1B gene located on chromosome 9 [3]. It has a reported incidence of 22 per million in the United Kingdom and 4.5 per million in the United States of America [4,5]. The first description of NPS has been credited to Chatelaine in 1820, when he initially described a triad of patella, nail and elbow involvement [6], with Turner later describing the pathognomonic iliac horns in 1933 [7]. Since the original description a more detailed understanding of the features of NPS has been elucidated. The most commonly seen clinical features are iliac horns (seen in 80% of cases), nail hypoplasia or aplasia, absent or hypoplastic patellae – with varying degrees of subluxation and radial head subluxation or dislocation [8]. Renal involvement has been reported in up to 42% of cases with asymptomatic proteinuria being the most common renal abnormality, only rarely progressing to renal failure [9]. Other less common features include scapula hypoplasia, scoliosis, genu valgum, club feet, heterochromia and glaucoma. The extent and severity of these features varies substantially between each individual.

NPS usually manifests as a benign condition, requiring only infrequent renal and ophthalmology observation. However, certain features can cause functional impairment. Up to 74% of cases display recurrent subluxation or dislocation of the patellae, leading to chronic pain and disruption of the extensor mechanism of the knee, often exacerbated by exercise [10].

Recognized long term complications of patella fractures include arthritis, muscle weakness and chronic pain. Interestingly, there is some recent limited evidence to suggest that the BMD of patients with NPS is lower, when compared to age matched controls [11]. Our patient exhibited mild osteopenia on DEXA scan and this may have been a contributory factor to the fracture. However, future studies with larger numbers are necessary to further evaluate decreased BMD with NPS.

While there are no data on the outcome of patella fractures in individuals with NPS it can be hypothesized that given the extent of pre-existing knee joint impairment in these individuals, functional outcome in this group may be inferior, suggesting the need for more frequent follow up.

## Conclusions

While NPS is a relatively rare condition, it has been extensively studied with follow up periods of up to 55 years [8]. Despite this, we believe the injury we present of a patella fracture in an individual with this condition is the first case of its kind to be reported in the literature.

## Consent

Written informed consent was obtained from the patient for publication of this case report and any accompanying images. A copy of the written consent is available for review by the Editor-in-Chief of this journal.

## Competing interests

The authors declare that they have no competing interests.

## Authors’ contributions

SCON analyzed and interpreted the patient data and wrote the manuscript. CGM contributed the surgical section of the manuscript and aided in the editorial process. JPMcE contributed to the preparation and aided in the editorial process. All authors read and approved the final manuscript.
